# Clinicopathological Risk Factors and Biochemical Predictors of Safe Discharge after Total Thyroidectomy and Central Compartment Node Dissection for Thyroid Cancer: A Prospective Study

**DOI:** 10.1155/2015/214525

**Published:** 2015-01-26

**Authors:** Yu-mi Lee, Ja Young Cho, Tae-Yon Sung, Tae Yong Kim, Ki-Wook Chung, Suck Joon Hong, Jong Ho Yoon

**Affiliations:** ^1^Department of Surgery, Asan Medical Center, University of Ulsan College of Medicine, Seoul 138-736, Republic of Korea; ^2^Department of Internal Medicine, Asan Medical Center, University of Ulsan College of Medicine, Seoul 138-736, Republic of Korea

## Abstract

To determine the clinicopathological risk factors and reliable biochemical predictors of the development of hypocalcemic symptoms after total thyroidectomy on the basis of serum calcium and intact parathyroid hormone (PTH) levels measured 1 hour after surgery, a prospective study was performed on 817 patients who underwent a total thyroidectomy with central compartment node dissection (CCND) due to well-differentiated thyroid cancer. We evaluated the correlations between hypocalcemic symptom development and clinicopathological factors. And the predictability for hypocalcemic symptom development of intact PTH cut-offs (<10 pg/mL and <20 pg/mL, resp.) according to serum calcium level subgroup was analyzed. Female gender (*P* < 0.001) was the only independent risk factor for hypocalcemic symptom development in multivariate regression analysis. The negative predictive value (NPV) of intact PTH, signifying nondevelopment of hypocalcemic symptoms, was higher than the positive predictive value (PPV) which signified development of hypocalcemic symptoms. In addition, when we applied the different adoption of the intact PTH cut-off according to serum calcium level, we could obtain more increased NPVs. A female gender and the application of more specific cut-offs for intact PTH according to the serum calcium levels measured 1 hour after surgery may help the patients to be more safely discharged.

## 1. Introduction

Despite every effort by endocrine surgeons to preserve the parathyroid glands during thyroid surgery, postoperative hypoparathyroidism is still one of the most common and sometimes most severe complications of a total thyroidectomy. Previous studies reported that the incidences of transient hypoparathyroidism were up to 83.0% and the incidences of permanent hypoparathyroidism ranged from 0.5% to 2% [1, 2]. Various clinicopathological profiles that increase the complexity of surgery, including large goiter, advanced thyroid carcinoma, bilateral thyroid surgery with concomitant cervical lymph node dissection, and reoperation, have been shown to increase the risk of hypoparathyroidism [3–9].

Although hypoparathyroidism affects the homeostasis of calcium, biochemical hypoparathyroidism does not always correspond with symptomatic hypoparathyroidism [4, 5, 10]. Patients with symptomatic and biochemical hypoparathyroidism require oral or intravenous calcium and/or vitamin D supplementation and they may often progress to permanent hypocalcemia. On the other hand, asymptomatic and only biochemical hypoparathyroidism is generally known that does not cause permanent hypocalcemia. Most of these patients do not need medication. Besides considering the lower life quality of patients with hypocalcemic symptoms, the most important factor affecting the postoperative course of total thyroidectomized patients would be whether hypocalcemic symptoms develop.

Prediction of the development or nondevelopment of hypocalcemic symptoms is very important for patients' safe discharge, because most total thyroidectomized patients are discharged either on the day of surgery or the day after surgery, recently. However, to date, the optimal protocol for the prediction of the development or nondevelopment of hypocalcemic symptoms is controversial. Herein, we performed a prospective study to identify patients at high or low risk of the development of hypocalcemic symptoms after total or completion total thyroidectomy and concurrent central compartment node dissection (CCND) in patients with well-differentiated thyroid cancer and to establish the ideal strategy for patients' safe discharge. Our hypothesis was that the cut-off of intact parathyroid hormone (PTH) level as a predictor of the development or nondevelopment of hypocalcemic symptoms should be adopted differently according to serum calcium level.

## 2. Materials and Methods

### 2.1. Demographics

1085 consecutive patients who underwent total thyroidectomy with or without CCND at Asan Medical Center between March 2008 and December 2010 were prospectively enrolled in this study. The study protocol was approved by our institutional review board. We excluded patients who had benign pathologies, who underwent lateral neck node dissection, or who did not undergo CCND. The patients with abnormal preoperative laboratory findings, including serum albumin, total calcium, alkaline phosphatase, intact PTH, or 25-OH vitamin D levels, were excluded. A total of 817 patients who underwent total thyroidectomy with CCND due to well-differentiated thyroid cancer were enrolled in this study, finally.

### 2.2. Clinicopathological Characteristics and Surgical Strategy

The clinicopathological characteristics of enrolled patients were evaluated, including age, gender, tumor size, types of cervical lymph node dissection, multiplicity, bilaterality, perithyroidal soft tissue extension, and status of lymph node metastasis (N classification). All operations were performed by a single endocrine surgeon (Yoon JH). Patients who were diagnosed with thyroid carcinoma on preoperative ultrasonography-guided fine needle aspiration cytology underwent a concomitant CCND, either ipsilateral or bilateral. A neck dissection was considered prophylactic when there was no evidence of lymphadenopathy on preoperative ultrasonography or intraoperative assessment of the central compartment.

Every effort was made to identify and preserve all parathyroid glands. However, if the parathyroid glands were not found in their usual location, no attempt was made to actively search for those glands that might be located far from the operative field. We thought that this might have subjected the parathyroid glands to unnecessary insult. If a parathyroid gland was devascularized or excised inadvertently, the gland was fragmented and immediately autotransplanted into the ipsilateral sternocleidomastoid muscle.

### 2.3. Postoperative Monitoring of Patients

All patients had their serum calcium and intact PTH levels checked 1 hour after surgery completion. Routine oral calcium and vitamin D supplements were not provided in this study. Calcium supplement was determined only by the presence of hypocalcemic symptoms, irrespective of serum calcium and intact PTH levels. Hypocalcemic symptoms included perioral or facial numbness, a tingling sensation or paresthesia of the hands and/or feet, positive Chvostek's or Trousseau's signs, muscular cramp, and tetany.

Patients were educated to report when these hypocalcemic symptoms develop and were examined by the medical staff. If patients' symptoms were identified as true hypocalcemic symptoms, the patients were prescribed 3.0 to 4.5 g of oral calcium carbonate and 1000 IU of vitamin D per day. Patients with severe symptoms despite oral calcium and vitamin D supplements were treated with additional intravenous calcium infusion.

All discharged patients were extensively counseled on the recognition and management of hypocalcemic symptoms. Patients who had experienced hypocalcemic symptoms had laboratory test recheck at the first postoperative visit (3 weeks after discharge). These patients were asked to stop the medication 2 days prior to a visit to the outpatient department. If there were no more hypocalcemic symptoms after cessation of medication and their serum calcium and intact PTH levels normalized, these patients were no longer prescribed the medications. On the other hand, if the hypocalcemic symptoms developed again, these patients were prescribed the medications again and followed up every 3 months. If the hypocalcemic symptoms were relieved after cessation of calcium and vitamin D supplements within 6 months after surgery, the hypoparathyroidism was classified as transient. In all other cases, it was classified as permanent hypoparathyroidism.

### 2.4. Laboratory Evaluations

Serum intact PTH levels were measured using an immunoradiometric assay (HAMILTON micorAP-2; Hamilton Company, Reno, NV) and determined from the same blood samples as the serum calcium. Serum calcium levels reported in this study were adjusted with patients' serum albumin levels and body mass index.

### 2.5. Study Design

Patients were categorized into two groups, symptomatic and asymptomatic, based on the presence of hypocalcemic symptoms and subdivided into three subgroups according to their serum calcium levels: ≤7.9 mg/dL, 8.0~8.5 mg/dL, and ≥8.6 mg/dL. The cut-off of intact PTH as a predictor of the development or nondevelopment of hypocalcemic symptoms was determined and calculated as 10 pg/mL and 20 pg/mL, respectively. First, the clinicopathological risk factors for the development of hypocalcemic symptoms were evaluated by univariate and multivariate regression analysis. Then, the predictability of intact PTH levels (10 pg/mL and 20 pg/mL) measured on the day of surgery for the development or nondevelopment of hypocalcemic symptoms was determined on the basis of serum calcium levels (≤7.9 mg/dL, 8.0~8.5 mg/dL, and ≥8.6 mg/dL).

### 2.6. Statistics

Statistical analysis was performed using a commercially available software package (SPSS version 13.0; SPSS Inc., Chicago, IL). The chi-square test was used for categorical variables and the paired two-tailed *t*-test was used for continuous variables. Furthermore, the Cox regression model was adopted for multivariate analysis. *P* values less than 0.05 were considered significant.

## 3. Results

### 3.1. Incidence of Hypocalcemic Symptoms

Among the enrolled patients, 345 (42.2%) developed hypocalcemic symptoms (Table 1). Hypocalcemic symptoms developed frequently even in patients with serum calcium levels ≥8.0 mg/dL (39.5%, 217/686). However, most of these patients had serum intact PTH less than 10 pg/mL (52.8%, 263/498) (Table 2). Permanent hypoparathyroidism was seen in only seven patients (0.7%).

### 3.2. Clinicopathological Risk Factors for Hypocalcemic Symptoms Development

In univariate regression analysis, younger patients (<45 years old; *P* = 0.025), female patients (*P* < 0.001), and patients who underwent bilateral CCND (*P* = 0.033) more frequently developed hypocalcemic symptoms. In multivariate regression analysis, female gender (odds ratio: 1.62; 95% confidence interval: 1.30~2.02; *P* < 0.001) was found to be the only independent risk factor for the development of hypocalcemic symptoms (Table 1).

Female patients also showed symptoms of hypocalcemia more frequently than male patients, when we compared the same serum calcium and intact PTH subgroups (Table 2). In the subgroups which had intact PTH ≥ 10 pg/mL, female patients had tendency to develop hypocalcemic symptoms compared to male patients, although the number of patients with hypocalcemic symptoms was too small to perform analysis. However, in the subgroups which had intact PTH < 10 pg/mL, the frequency of hypocalcemic symptoms was significantly higher in female patients than in male patients.

### 3.3. Biochemical Prediction of Hypocalcemic Symptom Development

We determined and calculated the cut-offs of intact PTH as a biochemical predictor of the development or nondevelopment of hypocalcemic symptoms as <10 pg/mL and <20 pg/mL, respectively, according to the three serum calcium subgroups (≤7.9 mg/dL, 8.0~8.5 mg/dL, and ≥8.6 mg/dL) (Table 3). All subgroups had high sensitivity, signifying development of hypocalcemic symptoms, but their specificities showed low values. Based on each intact PTH cut-off, its negative predictive value (NPV), signifying nondevelopment of hypocalcemic symptoms, was generally higher than its positive predictive value (PPV), signifying the possibility of the development of hypocalcemic symptoms. In the 8.0~8.5 mg/dL and ≥8.6 mg/dL serum calcium subgroups, the NPVs of intact PTH < 20 pg/mL were higher than those of intact PTH < 10 pg/mL. Besides, among the patients with intact PTH < 10 or 20 pg/mL, the ≥8.0 mg/dL serum calcium subgroups had higher NPVs than the ≤7.9 mg/dL.

## 4. Discussion

We here report our results of a prospective study to predict the development or nondevelopment of hypocalcemic symptoms after total thyroidectomy with CCND in patients with well-differentiated thyroid cancer. On the basis of our findings, female gender is an independent risk factor for the development of hypocalcemic symptoms (odds ratio: 1.62; 95% confidence interval: 1.30–2.02; *P* < 0.001). Furthermore, even in the same range of serum calcium and intact PTH levels, female patients feel hypocalcemic symptoms more frequently than male patients (*P* < 0.001). To our knowledge, this is the first report to describe a difference in the development of hypocalcemic symptoms according to sex. Hence, this gender difference may be considered an important risk factor when establishing a discharge plan for total thyroidectomized patients.

We also found that the intact PTH level measured on the day of surgery (1 hour after surgery) may be a valuable biochemical marker for predicting the nondevelopment of hypocalcemic symptoms, resulting in patients' safe early discharge. However, the intact PTH cut-offs should be adopted differently on the basis of serum calcium levels to minimize missing a delayed development of hypocalcemic symptoms.

With the increasing preference for shorter hospital stays, measurement of intact PTH alone or in combination with other biochemical parameters after total thyroidectomy has been used recently as a predictor of postoperative hypoparathyroidism [2, 5, 11–13]. In most previous studies [2, 3, 6, 7, 14–19], the predictive ability of serum intact PTH alone, using either a quick or a standard intact PTH assay, has been reported to be considerable, with a sensitivity ranging from 64% to 100% and a specificity of 72% to 100%. Both absolute levels and percent decline have also been reported to be used with similar accuracy. However, some study series recommended abandoning the use of postoperative intact PTH alone for predicting postoperative hypoparathyroidism. Lombardi et al. [20] concluded that intact PTH lacked the accuracy needed to predict hypoparathyroidism. In their large series of 523 consecutive patients who underwent total thyroidectomy, they found normal intact PTH levels at 4 postoperative hours in 70 hypocalcemic patients (13.4% false negative results), 11 of whom were symptomatic. They concluded that intact PTH alone did not accurately predict clinically relevant postoperative hypocalcemia and that the optimal cut-offs of intact PTH considering serum calcium levels should be needed. In another large series in which del Rio et al. [21] examined 1006 patients undergoing thyroidectomy, the authors concluded that a single measurement of intact PTH level 24 hours after thyroidectomy was not predictive of hypocalcemia. Intact PTH levels at 24 hours were within normal limits in 52 of these 101 patients (false negative rate of 20.6%). Moreover, another review of 458 patients undergoing thyroidectomy also found that 7% of patients with normal intact PTH levels developed hypocalcemia [1].

Recent studies have validated the role of postoperative intact PTH measurement in combination with serum calcium levels in predicting postthyroidectomy hypocalcemia and preventing its symptoms [3, 5]. One study stated that combined measurement of intact PTH levels (<15 pg/mL) on postoperative day 1 and serum calcium levels (<1.9 mmol/L) on postoperative day 2 had a remarkable performance (sensitivity, 96.3%; specificity, 96.1%; PPV, 86.0%; and NPV, 99.0%) in determining postthyroidectomy hypocalcemia [5]. Another study also demonstrated that changes in combined intact PTH and calcium levels 1 to 6 hours after thyroidectomy were accurate in predicting postoperative hypocalcemia [3]. In this latter study, a decline in serum intact PTH levels by more than 60%, in conjunction with a decline in serum calcium levels of more than 10%, 5 to 6 hours postoperatively had the best results (a sensitivity, specificity, and positive predictive value of 100%) for identifying patients at high risk of developing symptomatic hypocalcemia after total thyroidectomy [3].

When designing our study, our primary end point was the presence of hypocalcemic symptoms, irrespective of serum calcium or intact PTH levels. There were two reasons for focusing on hypocalcemic symptoms. One reason was that, as mentioned previously, asymptomatic and only biochemical hypoparathyroidism is not usually known to cause permanent hypocalcemia, and these patients were reported to regain normocalcemia after an average of 2.5 postoperative days without any need for calcium supplements [4, 5, 10]. The other reason was that we did not have quick intact PTH assay kits, so we could only confirm the intact PTH levels at several days after sampling (the result was reported only three times a week in our institution). We knew that this fact might be an important limitation of our study. We considered that the optimal timing of sampling should be as soon as possible after surgery because most patients were being discharged on the day of surgery or the day after surgery. Therefore, we decided on serum calcium and intact PTH levels obtained 1 hour after surgery as biochemical markers. We also regarded that any single biochemical marker might not be enough for accurately predicting the development or nondevelopment of hypocalcemic symptoms. Therefore, the cut-off of intact PTH as a predictor of the development or nondevelopment of hypocalcemic symptoms might be adopted differently according to serum calcium subgroup.

Though specificities of intact PTH cut-off were relatively low, all sensitivities of intact PTH were very high in our study. These results were in agreement with those of other studies that showed that the intact PTH cut-off may be a valuable predictor of the development of hypocalcemic symptoms. However, our result which different adoption of the intact PTH cut-off according to serum calcium level may increase the NPV is differentiated from previous reports. Among the patients with similar PTH results, the ≥8.0 mg/dL serum calcium subgroups had higher NPV than the <8.0 mg/dL serum calcium subgroup. In the 8.0~8.5 mg/dL and ≥8.6 mg/dL serum calcium subgroups, the NPVs of intact PTH <20 pg/mL were higher than those of intact PTH <10 pg/mL. Therefore, different adoption of the cut-off of intact PTH according to serum calcium levels enables patients who undergo a total thyroidectomy and CCND to be more safely discharged without calcium and vitamin D supplementation.

## 5. Conclusions

Female gender is an independent risk factor for the development of hypocalcemic symptoms in the patients with thyroid cancer. Furthermore, even in the same range of serum calcium and intact PTH levels, female patients feel hypocalcemic symptoms more frequently than male patients. This clinicopathological risk factor combined with more specific cut-offs for intact PTH, based on the serum calcium levels measured 1 hour after surgery, may help thyroid cancer patients who undergo a total thyroidectomy with CCND to be discharged more safely.

## Figures and Tables

**Table 1:** Univariate and multivariate clinicopathological risk factor analysis for the development of hypocalcemic symptoms.

Variables	Asymptomatic 472 (42.2%)	Symptomatic 345 (57.8%)	Univariate		Multivariate	
			OR (95% CI)	*P*	OR (95% CI)	*P*
Age						
<45 years	99 (21.0%)	96 (27.8%)	Ref.		Ref.	
≥45 years	373 (79.0%)	249 (72.2%)	0.69 (0.49~0.96)	0.025	0.84 (0.68~1.03)	0.096
Gender						
Male	111 (23.5%)	36 (10.4%)	Ref.		Ref.	
Female	361 (76.8%)	309 (89.6%)	2.63 (1.74~4.08)	<0.001	1.62 (1.30~2.02)	<0.001
Size						
≤1 cm	287 (60.8%)	208 (60.3%)	Ref.		Ref.	
>1 cm	185 (39.2%)	137 (39.7%)	1.02 (0.76~1.37)	0.885	1.02 (0.84~1.22)	0.870
Extent of CCND						
Unilateral	228 (48.3%)	140 (40.6%)	Ref.		Ref.	
Bilateral	244 (51.7%)	205 (59.4%)	1.36 (1.02~1.83)	0.033	1.16 (0.98~1.38)	0.080
Multifocality						
Unifocal	268 (56.8%)	174 (50.4%)	Ref.		Ref.	
Multifocal	149 (31.6%)	116 (33.6%)	1.2 (0.87~1.65)	0.269	1.11 (0.87~1.42)	0.400
Bilaterality						
Unilateral	318 (67.4%)	211 (61.2%)	Ref.		Ref.	
Bilateral	96 (20.3%)	66 (19.1%)	1.04 (0.71~1.50)	0.855	0.93 (0.70~1.23)	0.595
Perithyroidal extension						
Absent	181 (38.3%)	127 (36.8%)	Ref.		Ref.	
Present	291 (61.7%)	218 (63.2%)	1.07 (0.79~1.44)	0.662	0.97 (0.81~1.17)	0.767
N staging						
pN0	285 (60.4%)	204 (59.1%)	Ref.		Ref.	
pN1a	187 (39.6%)	141 (40.9%)	1.05 (0.79~1.41)	0.719	1.03 (0.87~1.24)	0.708

OR: odds ratio, CI: confidence interval, and Ref.: reference.

**Table 2:** Patterns in the development of hypocalcemic symptoms according to serum calcium and intact PTH levels.

Intact PTH (pg/mL)	Calcium (mg/dL)	Overall		Female		Male		*P* ^*^
		Number of patients	Symptomatic	Number of patients	Symptomatic	Number of patients	Symptomatic	
≤9.9	**≤7.9**	102	71 (69.6%)	89	63 (70.8%)	13	8 (61.5%)	0.528
	**8.0~8.5**	321	179 (55.8%)	260	160 (61.5%)	61	19 (31.1%)	<0.001
	**≥8.6**	177	84 (47.5%)	139	76 (54.7%)	38	8 (21.1%)	<0.001

10.0~19.9	**≤7.9**	22	2 (9.1%)	20	2 (10.0%)	2	0	NA
	**8.0~8.5**	91	5 (5.5%)	75	4 (5.3%)	16	1 (6.3%)	NA
	**≥8.6**	50	2 (4.0%)	40	2 (5.0%)	10	0	NA

≥20.0	**≤7.9**	7	1 (14.3%)	6	1 (16.7%)	1	0	NA
	**8.0~8.5**	34	1 (2.9%)	31	1 (3.2%)	3	0	NA
	**≥8.6**	13	0	10	0	3	0	NA

^*^Statistical difference in symptomatic hypocalcemia between female and male.

NA: not applicable.

**Table 3:** Predictabilities of intact PTH cut-offs according to serum calcium subgroup.

		All			Female			Male		
Calcium (mg/dL)		**≤7.9**	**8.0~8.5**	**≥8.6**	**≤7.9**	**8.0~8.5**	**≥8.6**	**≤7.9**	**8.0~8.5**	**≥8.6**

Number of patients		**131**	**446**	**240**	**115**	**366**	**189**	**16**	**80**	**51**

Intact PTH <10 (pg/mL)	Sensitivity	0.959	0.968	0.977	0.955	0.970	0.974	1.000	0.950	1.000
	Specificity	0.456	0.456	0.396	0.469	0.502	0.432	0.375	0.300	0.302
	PPV	0.696	0.558	0.475	0.708	0.615	0.547	0.615	0.311	0.211
	NPV	0.897	0.952	0.968	0.885	0.953	0.960	1.000	0.947	1.000
	Accuracy	0.740	0.668	0.604	0.748	0.713	0.656	0.688	0.463	0.412

Intact PTH <20 (pg/mL)	Sensitivity	0.986	0.995	1.000	0.985	0.994	1.000	1.000	1.000	1.000
	Specificity	0.105	0.126	0.084	0.102	0.149	0.090	0.125	0.050	0.070
	PPV	0.589	0.447	0.379	0.596	0.490	0.436	0.533	0.260	0.167
	NPV	0.897	0.971	1.000	0.833	0.968	1.000	1.000	1.000	1.000
	Accuracy	0.603	0.487	0.413	0.609	0.530	0.466	0.563	0.288	0.216
